# Effects of silicon nano-chelate on *Tetranychus urticae* populations and plant structural and physiological traits in rose cultivars

**DOI:** 10.1038/s41598-025-19979-4

**Published:** 2025-10-15

**Authors:** Roghayeh Hossein Hashemi, Hamed Aalipour, Ali Nikbakht, Nasim Aghamohammadi

**Affiliations:** 1 Department of Horticultural Science, Faculty of Agriculture, Tarbiat Modares University, Tehran, 1497713111 Iran; 2 Department of Landscape Engineering, Faculty of Agriculture, University of Tabriz, Tabriz, 5166616471 Iran; 3 Department of Horticulture, College of Agriculture, Isfahan University of Technology, Isfahan, Iran

**Keywords:** Biotic stress, Integrated pest management, Cell wall thickness, Nanoparticles, Insecticide, Two-spotted spider mite, Ecology, Ecology, Plant sciences

## Abstract

Roses are among the most economically important cut flowers globally, yet their production is severely impacted by two-spotted spider mites (*Tetranychus urticae*), which cause extensive damage and reduce marketability. This study evaluated the efficacy of silicon nano-chelate (N-Si) in suppressing mite populations and enhancing plant defenses in two rose cultivars, ‘Jumilia’ and ‘Samurai’, under controlled greenhouse conditions. A factorial experiment with six replicates was conducted. N-Si was applied at 2 mL L⁻¹ as a foliar spray every 10 days, while untreated plants served as controls. Results showed that N-Si significantly reduced mite populations, with egg, nymph, and adult counts declining by up to 67.5%, 98.4%, and 73.5%, respectively, after eight applications. Principal component analysis (PCA) revealed clear separation between treated and control samples, highlighting N-Si’s significant influence on both pest suppression and plant physiological traits. N-Si application increased cell wall thickness by 64.4%, contributing to mechanical resistance against mite feeding, with ‘Jumilia’ showing the highest structural reinforcement. Nutrient concentrations of silicon, calcium, and boron were significantly elevated in treated plants, particularly in ‘Samurai’. Photosynthetic pigments, including chlorophyll a, b, total chlorophyll, and carotenoids, increased by up to 28.3%, indicating improved physiological performance. Leaf area also expanded by 13.4% with N-Si, with ‘Jumilia’ exhibiting greater growth than ‘Samurai’. These findings demonstrate that N-Si enhances plant resilience through multiple defense mechanisms while effectively managing spider mite infestations, offering a sustainable, eco-friendly alternative to synthetic pesticides in rose cultivation.

## Introduction

Roses are among the top three most popular cut flowers globally and are highly sought after in international markets^1,2^. However, rose production faces significant challenges from two-spotted spider mites, which can reduce flower yield, cause aesthetic damage to leaves, and lead to substantial economic losses^3^. These pests can inflict damage ranging from 28% to 95% on rose plants^4^.

The two-spotted spider mite (*Tetranychus urticae* Koch.) is a major agricultural pest^5^ that causes extensive damage to a wide range of greenhouse ornamentals and vegetables worldwide. Greenhouse conditions, such as heating, cooling, and lighting, significantly influence their development and reproduction^6^. From the larval stage to adulthood, these mites primarily inhabit the abaxial (lower) surfaces of leaves, feeding by piercing leaf tissues with their stylets and extracting nutrients from the host plant^7,8^. Their feeding damages mesophyll cells, leading to the deterioration of chloroplasts, reduced chlorophyll content, and a decline in the net photosynthetic rate of plants^8,9^. Feeding symptoms include numerous small white or yellow spots on the leaf surface^10^, and at high infestation levels, they produce visible silk webbing^11^. Spider mites have an exceptionally high reproductive capacity, laying up to 7–10 eggs per female per day under optimal conditions. To maintain mite populations below the economic injury level, it is essential to reduce the number of viable eggs^12,13^. While acaricides are necessary when mite populations exceed economic thresholds^14^, the overuse of pesticides has led to environmental pollution and resistance issues, increasing the demand for eco-friendly and safe alternative control methods^7,15^.

The rapid advancement of nanotechnology offers new opportunities to improve pesticide formulations and safety through controlled release mechanisms on crop leaf surfaces and precise delivery of active ingredients^16,17^. These innovations reduce the effective dosage by maintaining optimal concentrations in target areas for extended periods, enhance penetration, and facilitate penetration across the leaf cuticle, making them a key focus in nano-agricultural applications^16–18^. Silicon (Si) nanoparticles, in particular, have garnered significant attention as pesticide carriers due to their low cost, porous nature, non-toxicity, high surface area, and reactivity^16,19,20^. Si is one of the most abundant elements in soil and is absorbed by plants in the form of silicic acid (Si (OH)₄)^21^. It is now recognized as a physical barrier when deposited beneath leaf cuticles, playing a critical role in enhancing plant tolerance to biotic stressors such as insect and mite pests, as well as plant diseases^22^. Si deposition increases the rigidity and abrasiveness of plant tissues, which deters chewing insects by wearing down their mouthparts^23,24^. Additionally, Si treatment has been shown to upregulate defense-related genes^22,25,41^, leading to increased activity of defensive enzymes and higher accumulation of defensive compounds, thereby reducing pest populations and damage^22^.

Despite these advancements, limited research has been conducted on nanoparticle-based pesticide delivery systems. Therefore, this study aimed to evaluate the efficacy of silicon nano-chelate (N-Si) in mitigating infestations of the two-spotted spider mite in two commercially important rose cultivars, ‘Jumilia’ and ‘Samurai’, under controlled greenhouse conditions. The cultivars ‘Jumilia’ and ‘Samurai’ were selected for this study due to their widespread commercial cultivation and their suspected differential susceptibility to mite infestation based on preliminary observations and grower reports. We hypothesized that foliar application of N-Si would significantly reduce mite populations by enhancing physical and biochemical plant defenses. Specifically, we predicted that N-Si would: suppress mite development and reproduction through mechanical and nutritional interference; increase cell wall thickness and silicon deposition, thereby reducing mite feeding success; improve photosynthetic performance via enhanced chlorophyll content and leaf area; and modulate nutrient uptake, particularly calcium and boron, contributing to overall plant resilience. Additionally, we expected cultivar-specific differences in response to N-Si treatment, based on observed physiological and structural variations between ‘Jumilia’ and ‘Samurai’.

## Materials and methods

### Experimental design and treatments

The study was conducted in the rose research greenhouses at Isfahan University of Technology (IUT), Isfahan, Iran (39°43’N, 51°32’E; 1683 m above sea level), from May to September 2022. Two rose cultivars, ‘Jumilia’ and ‘Samurai’, were grown hydroponically from grafted cuttings in a controlled-climate greenhouse compartment. The seedlings were transplanted into a wide, raised cultivation trough system, a standard setup for commercial rose production. The troughs were filled with a growing medium consisting of a 3:2 (v/v) mixture of cocopeat and perlite. Plants were supplied with a modified Hoagland’s nutrient solution (pH 5.5–5.8) containing the following concentrations per 1000 L of water: N: 50 g, P: 20.4 g, K: 59.7 g, Ca: 17.5 g, Mg: 6 g, Fe: 3.8 mg, Zn and Mn: 0.45 mg each, and Cu, Mo, and B: 0.15 mg each. The nutrient solution was applied daily for four minutes every hour.

During the experiment, the greenhouse conditions were maintained at 30–32°C during the day and 20–22°C at night, with a relative humidity of 38–40%. The study employed a factorial experiment arranged in a completely randomized design with two factors and six replications. The factors included two rose cultivars (*Rosa hybrida* cv. ‘Jumilia’ and ‘Samurai’) and foliar spray of N-Si at 2 mL L⁻¹, with untreated plants as controls. The N-Si solution was applied every 10 days, totaling 12 applications. The prepared solution was mixed with foliar spray soap at a concentration of 0.5 mL L⁻¹, and 250–300 mL of the solution was applied per plant. The N-Si fertilizer (Khazra, Sodour Ahrar Shargh Co., Iran) was characterized using Scanning Electron Microscopy (SEM). SEM analysis (HITACHI SU-5000 device, 500 kV, Research Institute of Petroleum Industry) confirmed the nanoscale morphology and showed particle sizes in the range of 40–50 nm^2^. The N-Si was supplied by Sodour Ahrar Shargh Company (Tehran, Iran), with a particle size of 40 nm (MH N. Chelate compounds. Google Patents, 2012). To manage other common greenhouse pests (e.g., aphids, powdery mildew), all plants were uniformly washed every three days with an ionic surfactant consisting of potassium salts of fatty acids (Jonoobgan, Tehran, Iran) at a concentration of 0.5 mL L⁻¹. This washing regimen was applied uniformly to all plants (control and treated) to manage other common greenhouse pests without known acaricidal effects on *T. urticae*. This practice is standard in commercial rose production in the region. While it may have a minor suppressive effect on mite populations, it was a constant factor across all treatments, allowing for a valid comparison of the N-Si effect.

### Evaluation of egg, nymph, and adult insect populations

**Fig. 1:**
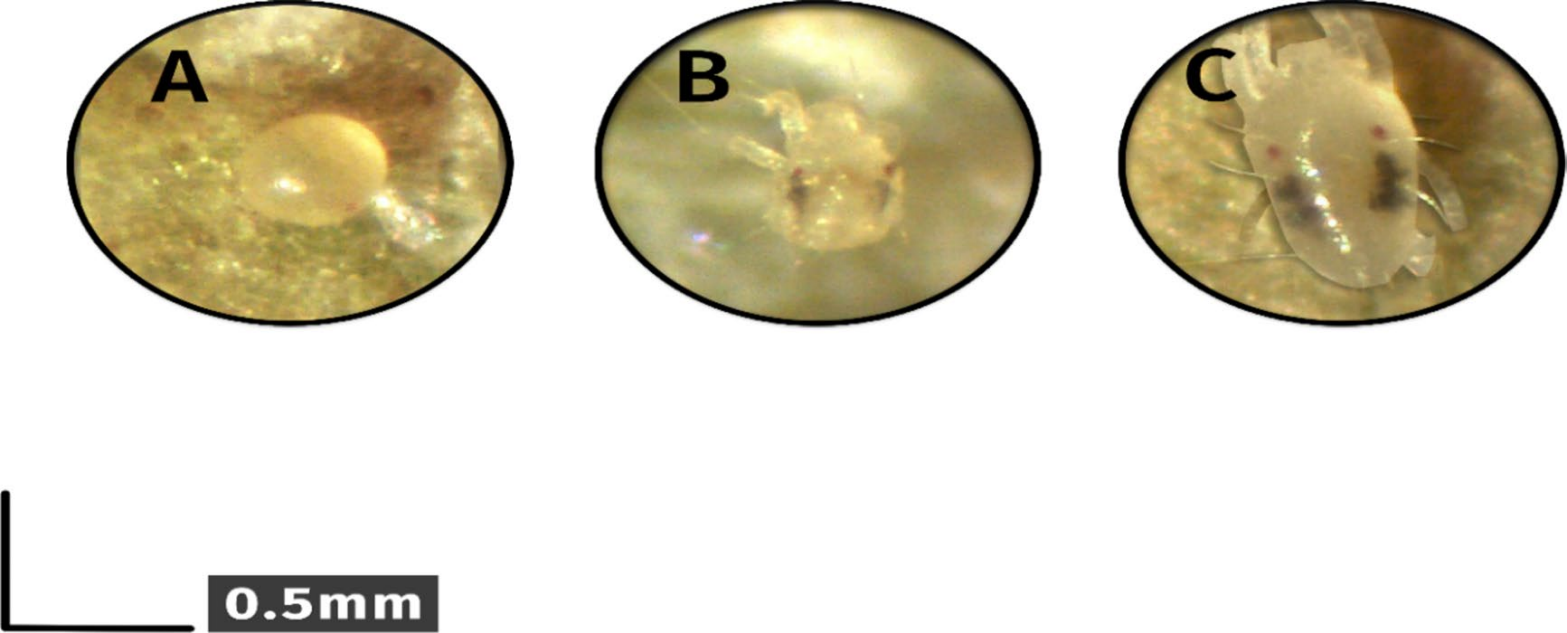
Two-spotted spider mites in three stages of the life cycle on cut rose flowers (**A**) egg, (**B**) nymphs, (**C**) adult mites.

The populations of eggs, nymphs, and adult mites were counted 30 cm above the flowering stem^3^ using binoculars (OSK 1844-B, Tokyo, Japan) after 4 and 8 applications of N-Si foliar spray. Six replications were performed per plant. Nymphs were categorized into two age groups (1 and 2), and both were collectively referred to as nymphs in this study (Fig. 1). Imaging was conducted using a binocular microscope with a 40× zoom and a Dino-Eye eyepiece camera, utilizing Dino Capture 2 software.

### Cell wall thickness

The first 5-leaflet leaf was collected after 12 applications of N-Si foliar spray and fixed in a 1:1:1 mixture of ethanol, water, and glycerin. Transverse sections of the leaf were stained sequentially with javel water (NaOCl), 1% acetic acid, carmine, and methyl green for 60, 30, 900, and 30 s, respectively. The sections were then mounted on slides using glycerin jelly^26^. Images were captured using an Olympus CH2 microscope equipped with a Dino-Eye eyepiece camera and Dino Capture 2 software.

### Nutrient concentrations

The concentrations of silicon (Si), calcium (Ca), and boron (B) were determined after 12 applications of N-Si foliar spray. Samples were oven-dried at 65 °C for 48 h, ground, and subjected to dry ashing at 550 °C for 5.5 h. The mineral composition was extracted using 2 N HCl and analyzed using inductively coupled plasma atomic emission spectrometry (ICP-OES, Varian Vista Pro, axial mode, Varian, Australia).

### Pigment concentrations

Photosynthetic pigment contents in the first 5-leaflet leaf were determined after 12 applications of N-Si foliar spray using the method described by Lichtenthaler (1987). Fresh leaf tissue (0.5 g) was homogenized in 10 mL of 100% acetone using a mortar and pestle. The absorbance of the supernatant was measured at 663 nm, 645 nm, and 450 nm using a spectrophotometer (V-160 A UV-Visible Recording Spectrophotometer, Shimadzu, Tokyo, Japan)^27^.

### Leaf area measurement

After 12 applications of N-Si foliar spray, 10 leaves were selected and scanned using a flatbed scanner (CanoScan LIDE 700 F). The leaf surface area was determined using Digimizer image analysis software, Version 4.1.1.0^28^.

### Statistical analysis

The experiment was conducted using a factorial design in a completely randomized layout with two factors: rose cultivars (‘Samurai’ and ‘Jumilia’) and N-Si concentrations (0 and 2 mL L⁻¹), each with six replications. Data normality was assessed using the Shapiro-Wilk test, and log transformations were applied if necessary. Analysis of variance (ANOVA) and mean comparisons were performed using the least significant difference (LSD) test at a significance level of P < 0.05, implemented in SAS software (Version 9.4, SAS Institute, Cary, NC, USA). Principal component analysis (PCA) was employed to assess the distribution of sample variance across principal components, with PC1 explaining 61.7% of the total variance and PC2 accounting for an additional 30.1%. This analysis revealed distinct patterns in the response of the cultivars to N-Si treatment, providing insights into the underlying mechanisms of mite population control and plant defense enhancement. PCA was conducted using Statgraphics software (Version 19).

## Result

### Evaluation of the egg, nymphs, and adult insect population

The application of nano-silicon (N-Si) significantly (*P* < 0.01) reduced the number of eggs, nymphs, and adult two-spotted spider mites at both stages (Table 1). After four applications of N-Si, the number of eggs and nymphs decreased by 67.5% and 80.5%, respectively, compared to the control (Table 3). After eight applications, the number of eggs, nymphs, and adult mites decreased by 56.7%, 98.4%, and 73.5%, respectively, compared to the control (Table 2). A significant cultivar × treatment interaction was observed (*P* < 0.05; Table 1). Results revealed that the lowest number of eggs and adult mites was recorded in treated ‘Jumilia’ plants, which was significantly different from treated ‘Samurai’ and control plants of both cultivars (Table 2).

**Table 1:** Effect of cultivar (Cul), foliar spray silicon nano-chelate (N-Si), on the number of eggs, nymphs and adult mites of two-spotted spider mites after 4 and 8 times of foliar spraying of two cut Rose flowers (cv. ‘Jumilia’ and ‘Samurai’).

Treatments	+ Means of two-spotted spider mite					
	Egg 1	Nymphs 1	Adult 1	Egg 2	Nymphs 2	Adult 2
**Cultivar**						
Cul1	87.4 ± 64^a^	7.55 ± 10.5^b^	6.66 ± 5.47 ^a^	1.94 ± 1.18^a^	0.500 ± 0.540^b^	0.796 ± 0.761^b^
Cul2	82.2 ± 50.7^a^	11.7 ± 10.4^a^	8.22 ± 2.09^a^	1.48 ± 0.818^b^	0.740 ± 0.872^a^	1.24 ± 1.06^a^
**Foliar spray status**						
Non-AP N-Si	128 ± 49.6^a^	16.1 ± 11.7^a^	8 ± 4.94^a^	2.38 ± 1.10^a^	1.22 ± 0.571^a^	1.61 ± 0.919^a^
AP N-Si	41.6 ± 20.1^b^	3.14 ± 2.37^b^	6.88 ± 3.24^a^	1.03 ± 0190^b^	0.018 ± 0.136^b^	0.425 ± 0.499^b^
**Significance**						
Cultivar (Cul)	ns	**	*	**	**	**
Silicon nano-chelate (N-Si)	**	**	ns	**	**	**
Cul × N-Si	ns	**	ns	**	**	ns
CV%	24.8	28.6	25.6	24.5	26.4	29.8

*Significant at *P* = 0.05.

**Significant at *P* = 0.01.

ns Non-significant.

+Mean separation with column by the Least Significant Difference test (LSD) at α = 0.01. Means in the same column followed by the same letters are not statistically different at *P* = 0.01.

+ Within each column means followed by the same letters do not express significant differences according to LSD at *P* < 0.05.

1 and 2 are the symbols of the foliar spray of nano-silicon after 4 times and 8 times, respectively.

**Table 2:** Interaction effects of the cultivar (Cul) × silicon nano-chelate (N-Si) on the number of eggs, nymphs and adult mites of two-spotted spider mites after 4 and 8 times of foliar spraying of two cut Rose flowers (cv. ‘Jumilia’ and ‘Samurai’).

Treatment		+ Mean square of two-spotted spider mite					
		Egg 1	Nymphs 1	Adult 1	Egg 2	Nymphs 2	Adult 2
Cul	N-Si						
‘Jumilia’	NA	122 ± 36.6^a^	20.4 ± 7.82^a^	8.62 ± 1.54^a^	1.92 ± 0.957^b^	1.44 ± 0.697^a^	1.85 ± 1.13^a^
	AP	41.9 ± 22.7^b^	3.14 ± 2.86^c^	7.81 ± 2.49^ab^	1.03 ± 0.192^c^	0.037 ± 0.192^c^	0.629 ± 0.492^c^
‘Samurai’	NA	133 ± 60.2^a^	11.9 ± 13.5^b^	7.37 ± 6.82^ab^	2.85 ± 1.06^a^	1 ± 0.277^b^	1.37 ± 0.564^b^
	AP	41.3 ± 17.5^b^	3.14 ± 1.81^c^	5.96 ± 3.66^b^	1.03 ± 0.192^c^	0 ± 0^c^	0.222 ± 0.423^d^

Within each column means followed by the same letters do not express significant differences according to LSD at *P* < 0.05.

1 and 2 are the symbols of the foliar spray of nano-silicon after 4 times and 8 times, respectively.

NA: Not application, AP: application.

**Table 3:** Effect of cultivar (Cul), foliar spray silicon nano-chelate (N-Si), on the wall thickness, silicon content (Si), Boron (B), calcium (Ca), chlorophyll a (Chl a), chlorophyll b (Chl b), total chlorophyll (Total Chl) and carotenoid (Car) content and leaf area of two cut Rose flowers (cv. ‘Jumilia’ and ‘Samurai’).

Treatments	+ Means of some measured parameters								
	Cell wall thickness	Si	B	Ca	Chl a	Chl b	Total Chl	Car	Leaf area
	mm	µg g^− 1^ DW		g kg-^1^ DW	g kg^− 1^ FW				cm^2^
**Cultivar**									
Cul1	0.009 ± 0.002^a^	0.546 ± 0.131^b^	13.5 ± 2.51^b^	1.03 ± 0.125^b^	17.2 ± 2.78^a^	7.69 ± 1.60^a^	24.9 ± 4.11^a^	16.7 ± 3.81^a^	158 ± 19.01^a^
Cul2	0.007 ± 0.003^a^	0.722 ± 0.134^a^	17.3 ± 2.62^a^	1.13 ± 0.063^a^	15.2 ± 2.58^a^	6.82 ± 1.91^a^	22.1 ± 4.36^a^	14.7 ± 4.38^a^	126 ± 14.7^b^
**Foliar spray status**									
Non-AP N-Si	0.006 ± 0.001^b^	0.547 ± 0.118^b^	16.4 ± 3.59^a^	1.05 ± 0.133^a^	15.06 ± 2.50^b^	6.43 ± 1.60^b^	21.5 ± 4.02^b^	13.8 ± 3.59^b^	133 ± 16^b^
AP Si	0.010 ± 0.002^a^	0.721 ± 0.142^a^	14.5 ± 2.24^a^	1.11 ± 0.069^a^	17.4 ± 2.57^a^	8.08 ± 1.52^a^	25.5 ± 3.71^a^	17.7 ± 3.66^a^	151 ± 26^a^
**Significance**									
Cultivar (Cul)	ns	**	**	**	ns	ns	ns	ns	**
Silicon nano-chelate (N-Si)	**	**	ns	ns	*	**	**	**	**
Cul × N-Si	ns	ns	ns	**	ns	*	ns	ns	ns
CV%	28.9	16.03	15.4	6.82	14.9	20.1	15.5	21.6	9.86

*Significant at *P* = 0.05.

**Significant at *P* = 0.01.

ns Non-significant.

+Mean separation with column by the Least Significant Difference test (LSD) at α = 0.01. Means in the same column followed by the same letters are not statistically different at *P* = 0.01.

+ Within each column means followed by the same letters do not express significant differences according to LSD at *P* < 0.05.

### Cell wall thickness

**Fig. 2:**
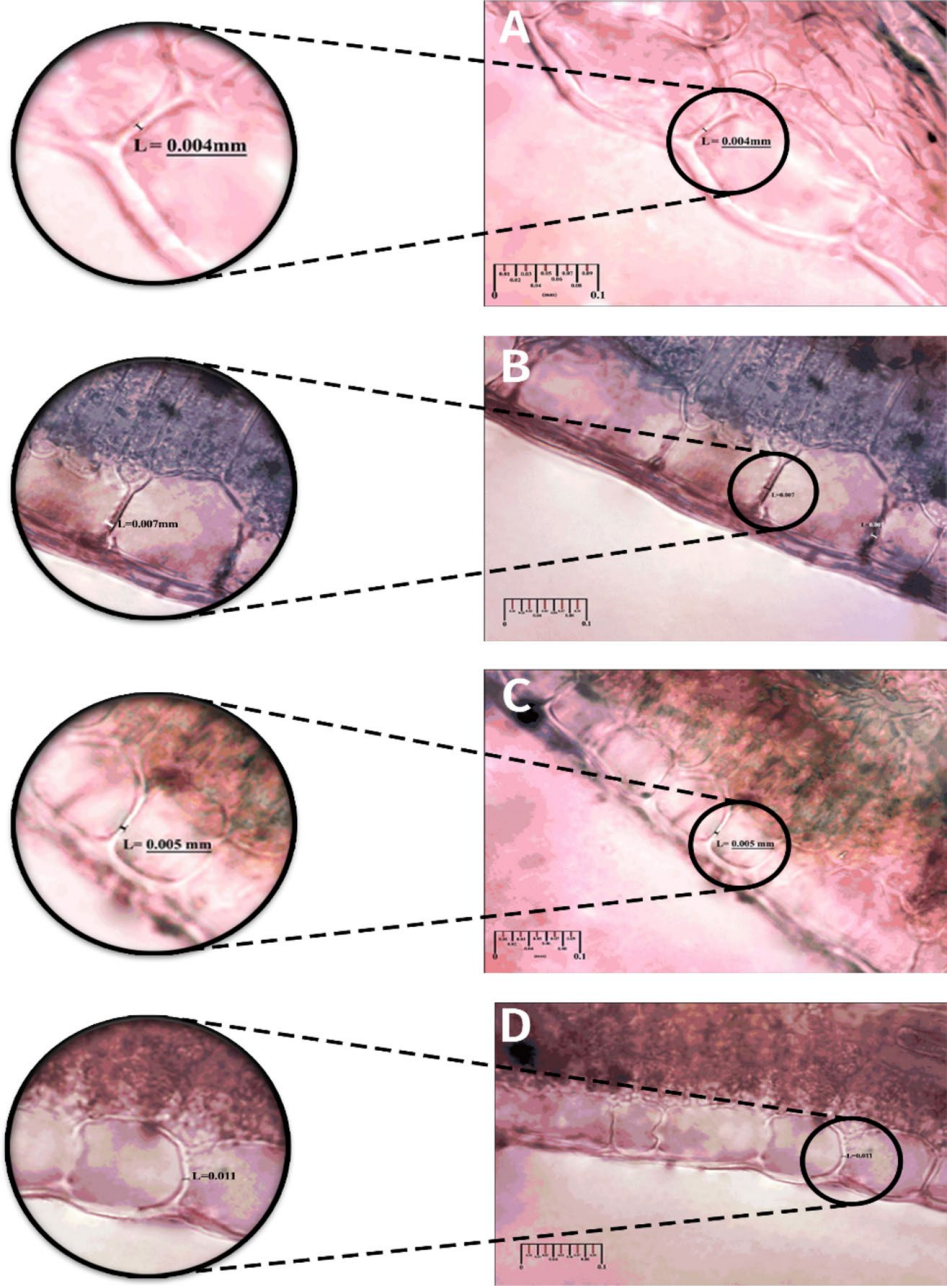
Light micrographs showing transverse sections of cell wall thickness of different rose cultivars in the first leaflet the 5-leaflet leaf of the flowering stem. (**A**) Effect of non-application of silicon nano-chelate (N-Si) in ‘Jumilia’ (control), (**B**) effect of application of N-Si in ‘Jumilia’, (**C**) effect of non-application of N-Si in ‘Samurai’ (control), (**D**) effect of application of N-Si in ‘Samurai’.

The application of N-Si significantly increased cell wall thickness at the 5% level (Table 3). Compared to the control, N-Si application resulted in a 64.4% increase in cell wall thickness. Additionally, ‘Jumilia’ exhibited a 22.2% greater cell wall thickness than ‘Samurai’ (Table 3). The highest cell wall thickness was observed in ‘Jumilia’ (0.011 mm) followed by ‘Samurai’ (0.009 mm) with N-Si application, while the lowest thickness was recorded in the control treatment of ‘Samurai’ (Table 4; Fig. 2).

**Table 4:** Interaction effects of the cultivar (Cul) × silicon nano-chelate (N-Si) on the cell wall thickness, silicon content (Si), Boron (B), calcium (Ca), chlorophyll a (Chl a), chlorophyll b (Chl b), total chlorophyll (Total Chl) and carotenoid (Car) content and leaf area of two cut Rose flowers (cv. ‘Jumilia’ and ‘Samurai’).

Treatment		+ Mean square of some measured parameters								
		Cell wall thickness	Si	B	Ca	Chl a	Chl b	Total Chl	Car	Leaf area
		mm	µg g^− 1^ DW		g kg-^1^ DW	g kg^− 1^ FW				cm^2^
Cul	N-Si									
‘Jumilia’	NA	122 ± 0.001^a^	0.636 ± 0.056^b^	18.9 ± 1.95^a^	1.17 ± 0.058^a^	13.5 ± 2.22^b^	5.37 ± 0.992^b^	18.9 ± 3.20^b^	11.4 ± 2.05^b^	121 ± 9.57^c^
	AP	41.9 ± 0.003^b^	0.808 ± 0.136^a^	15.8 ± 2.30^b^	1.10 ± 0.053^a^	17 ± 1.59^a^	8.27 ± 1.42^a^	25.2 ± 2.71^a^	18.09 ± 3.36^a^	130 ± 18.2^bc^
‘Samurai’	NA	133 ± 0.001^a^	0.459 ± 0.108^c^	13.9 ± 3.44^b^	0.943 ± 0.090^b^	16.5 ± 2.12^a^	7.49 ± 1.59^a^	24.08 ± 3.55^a^	16.1 ± 3.64^a^	145 ± 13.9^b^
	AP	41.3 ± 0.001^b^	0.634 ± 0.088^b^	13.2 ± 1.32^b^	1.12 ± 0.086^a^	17 ± 3.39^a^	7.89 ± 1.73^a^	25.7 ± 4.78^a^	17.3 ± 4.22^a^	171 ± 12.8^a^

Within each column means followed by the same letters do not express significant differences according to LSD at *P* < 0.05.

NA: Not application, AP: application.

### Nutrient concentrations

The contents of calcium (Ca), silicon (Si), and boron (B) in ‘Samurai’ and ‘Jumilia’ cut rose flowers were significantly influenced by N-Si application (P < 0.01) (Table 3). Compared to ‘Jumilia’, ‘Samurai’ exhibited increases of 10.33%, 1.32%, and 28.1% in leaf Ca, Si, and B content, respectively (Table 3). N-Si application increased leaf Si content by 31.6% compared to the control. The highest Si content was observed in ‘Samurai’ with N-Si application, while the highest B content was recorded in the control treatment of ‘Samurai’. The highest Ca content was found in ‘Jumilia’ with N-Si application (Table 4).

### Photosynthetic pigment concentrations

N-Si application significantly increased chlorophyll a, chlorophyll b, total chlorophyll, and carotenoid contents by 15.6%, 25.6%, 18.6%, and 28.3%, respectively, compared to the control (Table 3). The highest chlorophyll a, and total chlorophyll content were observed in ‘Jumilia’, while the highest chlorophyll b and carotenoid content were recorded in ‘Samurai’ following N-Si application (Table 4).

### Leaf area measurement

Leaf area was significantly influenced by both cultivar and N-Si application (P < 0.01) (Table 3). ‘Jumilia’ exhibited a 25.5% larger leaf area compared to ‘Samurai’, while N-Si application increased leaf area by 13.4% compared to the control (Table 3). The highest leaf area was observed in ‘Jumilia’ with N-Si application, while the lowest was recorded in the control treatment of ‘Samurai’ (Table 4).

### Principal component analysis (PCA)

**Fig. 3:**
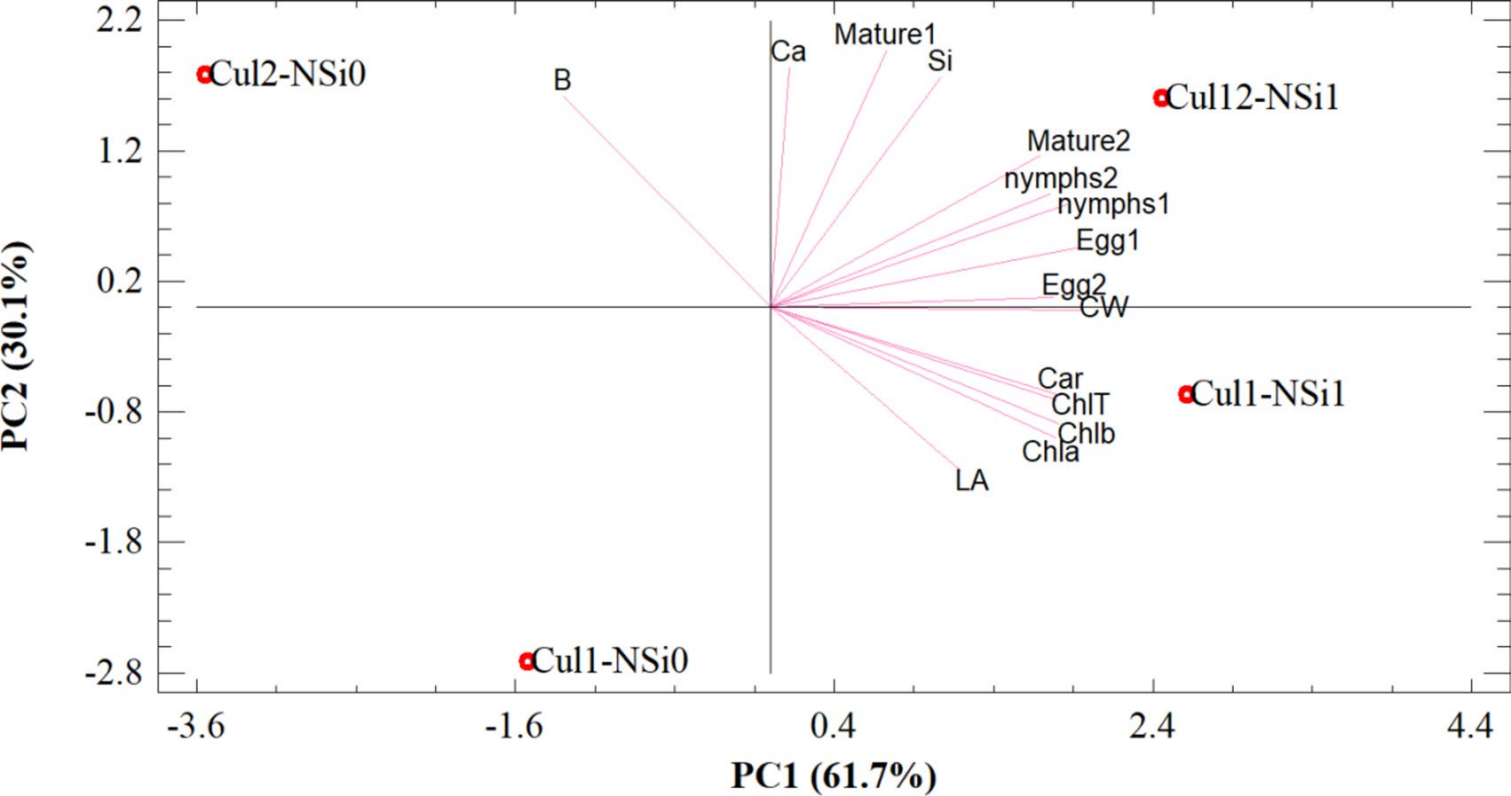
Principal Component Analysis (PCA) performed on mite population dynamics and plant defense characteristics in two rose cultivars (‘Jumilia’ and ‘Samurai’) treated with silicon nano-chelate (N-Si). Variables include mite life stages (Egg1, Egg2, nymph1, nymph2, Mature1, Mature2), leaf area (LA), cell wall thickness (CW), chlorophyll content (Chla, Chlb, ChlT), carotenoids (Car), calcium (Ca), and silicon (Si). Treatments: Cul1-Si0 and Cul2-Si0 (untreated controls); Cul1-Si1 and Cul2-Si1 (N-Si treated).

The PCA plot clearly demonstrates that the application of N-Si significantly influenced both mite population dynamics and plant physiological responses (Fig. 3). The first principal component (PC1), which captured the majority of the variance, primarily reflected differences in mite life stages, including eggs (Egg1, Egg2), nymphs (nymphs1, nymphs2), and mature mites (Mature1, Mature2). Samples treated with N-Si (labeled as Cul1-Si1 and Cul2-Si1) were distinctly separated from untreated controls (Cul1-Si0 and Cul2-Si0) along PC1, indicating a pronounced reduction in mite populations across all life stages. This suggests that N-Si effectively disrupted mite development and reproduction, thereby mitigating infestations in both rose cultivars.

The second principal component (PC2) highlighted variations in plant defense-related traits, such as leaf area, cell wall integrity, and chlorophyll content (Chla, Chlb, ChlT). Notably, N-Si-treated samples exhibited enhanced leaf area and improved chlorophyll levels, reflecting increased photosynthetic capacity and overall plant vigor. Additionally, the proximity of these samples to variables like “Cell wall” and “Si” on the PCA plot suggests that N-Si supplementation strengthened plant structural defenses, potentially through silicon deposition in cell walls. This reinforcement likely contributed to the observed resistance against mite infestations.

Further analysis revealed that the silicon nano-chelate formulation played a critical role in modulating calcium (Ca) uptake and distribution within the plants. The close association of “Ca” with N-Si-treated samples along PC2 indicates that N-Si facilitated calcium transport or accumulation, which is known to enhance plant defense responses. “Ca” is essential for activating defense pathways and strengthening cell walls, thereby complementing the direct effects of N-Si on mite populations.

Comparative analysis between the two rose cultivars (‘Jumilia’ and ‘Samurai’) showed that while both responded positively to N-Si treatment, ‘Samurai’ exhibited a more robust response, particularly in terms of mite population reduction and enhanced chlorophyll content. Comparative analysis of the PCA results between the two rose cultivars (‘Jumilia’ and ‘Samurai’) showed that while both responded positively to N-Si treatment, ‘Samurai’ exhibited a more robust response, particularly in terms of mite population reduction and enhanced chlorophyll content, as evidenced by its distinct clustering and variable loadings in the PCA biplot (Fig. 3).

## Discussion

The present study demonstrates that silicon nano-chelate (N-Si) is an effective strategy for mitigating two-spotted spider mite infestations while concurrently enhancing key physiological and structural defenses in rose cultivars ‘Jumilia’ and ‘Samurai’. Our central hypothesis is strongly supported by the significant reduction in mite populations across all life stages—eggs (56.7%), nymphs (98.4%), and adults (73.5%)—following N-Si application. These results align with established literature on the protective role of silicon against arthropod pests, particularly piercing-sucking herbivores like spider mites^29,30^.

The profound suppression of mite populations, especially the near-total reduction in nymphs, suggests N-Si disrupts multiple aspects of mite biology. While our study did not directly examine mite feeding structures, the proposed mechanism, well-documented in other plant-pest systems^23,24,31^, is that silicon deposition creates a physical abrasive barrier. We posit that this barrier likely increases the difficulty of stylet penetration, accelerates mouthpart wear, and reduces the suitability of leaf tissues for oviposition. This cumulative physical stress directly links to our observed population declines by potentially lowering feeding efficiency, increasing juvenile mortality, and impairing reproduction.

Our findings provide a direct connection between induced plant defenses and pest suppression. The N-Si-mediated increase in cell wall thickness (64.4%) is a prime example. This structural reinforcement, resulting from silicon polymerization into amorphous silica^32,33^, is not merely a parallel observation but a key factor likely responsible for the increased physical resistance to mite feeding. Thicker, more rigid cell walls can physically impede stylet insertion and reduce the accessibility of cell contents, thereby directly contributing to the reduced feeding success and subsequent population decline observed in our study.

Beyond mechanical defenses, N-Si application enhanced the plants’ physiological resilience, which may have indirectly compromised mite fitness. The significant increases in photosynthetic pigments (chlorophyll a, b, total chlorophyll, and carotenoids up 28.3%) and leaf area (13.4%) indicate a superior overall plant vigor. Healthier plants with robust photosynthetic apparatus are better equipped to tolerate and repair feeding damage. This enhanced tolerance likely reduces the net nutritional gain for the mites, potentially leading to poorer pest performance and lower reproductive rates. Furthermore, elevated carotenoids suggest improved photoprotection and reduced oxidative stress^34^, creating a less favorable biochemical environment for the pests. These findings are supported by previous studies showing that Si promotes chlorophyll synthesis by improving nutrient availability and preserving chloroplast structure^35,36^. The observed increases in leaf area further underscore the growth-promoting effects of N-Si, likely due to enhanced water-use efficiency, xylem transport, and turgor pressure mediated by Si nanoparticles^37,38^.

The synergy between different defense layers was further illuminated by the nutrient analysis. The significant elevation of leaf silicon, calcium, and boron indicates that N-Si improves nutrient uptake and utilization. Calcium is crucial for activating defense signaling pathways and fortifying cell walls^38,39^, while boron is integral to cell wall structure and function^40^. The association of these nutrients with N-Si treatment in the PCA plot suggests a multifaceted defense strategy: silicon provides the primary abrasive material, while calcium and boron support the structural integrity and signaling necessary for an effective defense response. This nutritional enhancement contributes to the overall plant resilience that underpins the observed mite suppression.

Cultivar-specific responses revealed intriguing genetic differences in defense strategy. ‘Samurai’ exhibited a more robust reduction in mite counts and higher levels of chlorophyll b and carotenoids, suggesting a physiology that is highly responsive to Si-induced vigor. In contrast, ‘Jumilia’ developed greater cell wall thickness and accumulated more calcium, indicating a predisposition towards structural fortification. This genotype-specificity highlights that the efficacy of N-Si is modulated by the plant’s inherent metabolic pathways and underscores the importance of tailoring silicon-based pest management strategies to specific cultivars.

In conclusion, our results demonstrate that N-Si application orchestrates a multi-layered defense response in rose plants. We propose a model whereby N-Si is absorbed and deposited, leading to: (1) Mechanical resistance through cell wall thickening, which physically impedes mites; (2) Physiological resilience through enhanced photosynthesis and leaf area, which improves tolerance to damage; and (3) Nutritional fortification that supports these defense structures and signals. Together, these changes create a hostile environment that directly and indirectly suppresses *T. urticae* development, feeding, and reproduction. This study firmly establishes silicon nano-chelate not just as a standalone treatment, but as a valuable component of an integrated, sustainable pest management program for rose cultivation, reducing reliance on conventional chemical acaricides. Future work should focus on the molecular mechanisms of this induced resistance and validate these promising results in long-term field trials.

## Conclusion

This study underscores the significant potential of silicon nano-chelate (N-Si) as an effective and sustainable solution for managing two-spotted spider mite infestations in rose cultivation. The application of N-Si not only reduced mite populations by up to 98.4% but also reinforced plant defenses through increased cell wall thickness and enhanced nutrient uptake, particularly silicon, calcium, and boron. The observed improvements in photosynthetic pigment concentrations and leaf area further demonstrate N-Si’s role in promoting plant growth and resilience. These findings align with previous research on silicon’s ability to enhance physical and biochemical defenses in plants, offering a viable alternative to chemical pesticides. By integrating N-Si into rose cultivation practices, growers can achieve improved pest management, enhanced plant health, and increased yield quality, contributing to more sustainable and eco-friendly agricultural practices. Future research should explore the long-term effects of N-Si application across different rose cultivars and its potential in integrated pest management systems.

## Data Availability

The datasets generated and analyzed during this study are available from the corresponding author (Roghayeh Hossein Hashemi) upon reasonable request.

## References

[1] Kono, A. et al. Control of black spot disease by Ultraviolet-B irradiation in Rose (*Rosa × hybrida*) production. *J. Hortic.* **92**, 88–96. 10.2503/hortj.QH-037 (2023).

[2] Hashemi, R. H., Nikbakht, A. & Aalipour, H. Synergistic effects of oxygen nanobubble, nano-silicon and seaweed extract on promoting quality and postharvest performance of two cut Rose flowers. *Sci. Hortic.* **338**, 113637. 10.1016/j.scienta.2024.113637 (2024).

[3] Zhang, Z. Q. & Sanderson, J. P. Two-spotted spider mite (Acari: Tetranychidae) and *Phytoseiulus persimilis* (Acari: Phytoseiidae) on greenhouse roses: Spatial distribution and predator efficacy. *J. Econ. Entomol.* **88**, 352–357. 10.1093/jee/88.2.352 (1995).

[4] Hegde, J. N., Ashrith, K. N., Suma, G. S., Chakravarthy, A. K. & Gopalkrishna, H. R. Insect pests of roses and their management. In *Advances in Pest Management in Commercial Flowers.*. 85–102. (Apple Academic Press, 2020). 10.4324/9780429284120-6

[5] Miresmailli, S. & Isman, M. B. Efficacy and persistence of Rosemary oil as an acaricide against two-spotted spider mite (Acari: Tetranychidae) on greenhouse tomato. *J. Econ. Entomol.* **99**, 2015–2023. 10.1093/jee/99.6.2015 (2006). 17195668 10.1603/0022-0493-99.6.2015

[6] Knapp, M., Palevsky, E. & Rapisarda, C. Insect and mite pests. *Integr. Pest Disease Manage. Greenh. Crops*. **9**, 101–146. 10.1007/978-3-030-22304-54 (2020).

[7] Attia, S. et al. A review of the major biological approaches to control the worldwide pest *Tetranychus urticae* (Acari: Tetranychidae) with special reference to natural pesticides: biological approaches to control *Tetranychus urticae*. *J. Pest Sci.* **86**, 361–386. 10.1007/s10340-013-0503-0 (2013).

[8] Akyazı, R. & Soysal, M. Effect of *Tetranychus urticae* and *Polyphagotarsonemus latus* (Acari: Tetranychidae, Tarsonemidae) at different infestation levels and feeding durations on chlorophyll content of bean plants. *Plant. Prot. Bull.* **64**, 5–13. 10.16955/bitkorb.1302239 (2024).

[9] Ziaee, M. & Nikpay, A. Effect of mite damage on chlorophyll content of commercial sugarcane varieties using SPAD meter. *J. Sugarcane Res.* **6**, 59–62 (2016).

[10] Park, Y. L. & Lee, J. H. Leaf cell and tissue damage of cucumber caused by twospotted spider mite (Acari: Tetranychidae). *J. Econ. Entomol.* **95**, 952–957. 10.1093/jee/95.5.952 (2002). 12403421 10.1093/jee/95.5.952

[11] Yanar, D., Kadıoglu, I. & Gokce, A. Ovicidal activity of different plant extracts on two-spotted spider mite (*Tetranychus urticae* Koch) (Acari: Tetranychidae). *Sci. Res. Essays*. **6**, 3041–3044 (2011).

[12] Chow, A., Chau, A. & Heinz, K. M. Reducing fertilization for cut roses: effect on crop productivity and two-spotted spider mite abundance, distribution, and management. *J. Econ. Entomol.* **102**, 1896–1907. 10.1603/029.102.0521 (2009). 10.1603/029.102.0521 19886455

[13] Van Leeuwen, T., Vontas, J., Tsagkarakou, A., Dermauw, W. & Tirry, L. Acaricide resistance mechanisms in the two-spotted spider mite *Tetranychus urticae* and other important Acari: a review. *Insect Biochem. Mol. Biol.* **40**, 563–572. 10.1016/j.ibmb.2010.05.008 (2010). 20685616 10.1016/j.ibmb.2010.05.008

[14] Jakubowska, M., Dobosz, R., Zawada, D. & Kowalska, J. A review of crop protection methods against the twospotted spider mite—*Tetranychus urticae* Koch (Acari: Tetranychidae)—with special reference to alternative methods. *Agriculture* **12**, 898. 10.3390/agriculture12070898 (2022).

[15] Van Leeuwen, T., Tirry, L., Yamamoto, A., Nauen, R. & Dermauw, W. The economic importance of acaricides in the control of phytophagous mites and an update on recent acaricide mode of action research. *Pestic Biochem. Physiol.* **121**, 12–21. 10.1016/j.pestbp.2014.12.009 (2015). 26047107 10.1016/j.pestbp.2014.12.009

[16] Magda, S. & Hussein, M. M. Determinations of the effect of using silica gel and nano-silica gel against Tutaabsoluta (Lepidoptera: Gelechiidae) in tomato fields. *J. Chem. Pharm. Res.* **8**, 506–512 (2016).

[17] Zhao, X. et al. Development strategies and prospects of nano-based smart pesticide formulation. *J. Agric. Food Chem.* **66**, 6504–6512. 10.1021/acs.jafc.7b02004 (2017). 28654254 10.1021/acs.jafc.7b02004

[18] Smith, K., Evans, D. A. & El-Hiti, G. A. Role of modern chemistry in sustainable arable crop protection. *Philos. Trans. R Soc. B Biol. Sci.* **363**, 623–637. 10.1098/rstb.2007.2174 (2008). 10.1098/rstb.2007.2174 PMC2610174 17702697

[19] Wen, L. X. et al. Controlled release of avermectin from porous hollow silica nanoparticles. *Pest Manag Sci. Former. Pestic Sci.* **61**, 583–590. 10.1002/ps.1032 (2005). 10.1002/ps.1032 15714463

[20] Xu, C. et al. Emulsion-based synchronous pesticide encapsulation and surface modification of mesoporous silica nanoparticles with carboxymethyl Chitosan for controlled azoxystrobin release. *Chem. Eng. J.* **348**, 244–254. 10.1016/j.cej.2018.05.008 (2018).

[21] Debona, D., Rodrigues, F. A. & Datnoff, L. E. Silicon’s role in abiotic and biotic plant stresses. *Annu. Rev. Phytopathol.* **55**, 85–107. 10.1146/annurev-phyto-080516-035312 (2017). 28504920 10.1146/annurev-phyto-080516-035312

[22] Reynolds, O. L., Padula, M. P., Zeng, R. & Gurr, G. M. Silicon: potential to promote direct and indirect effects on plant defense against arthropod pests in agriculture. *Front. Plant. Sci.* **7**, 188037. 10.3389/fpls.2016.00744 (2016). 10.3389/fpls.2016.00744 PMC4904004 27379104

[23] Massey, F. P. & Hartley, S. E. Physical defences wear you down: progressive and irreversible impacts of silica on insect herbivores. *J. Anim. Ecol.* **78**, 281–291. 10.1111/j.1365-2656.2008.01472.x (2009). 18771503 10.1111/j.1365-2656.2008.01472.x

[24] Alhousari, F. & Greger, M. Silicon and mechanisms of plant resistance to insect pests. *Plants* **7**, 33. 10.3390/plants7020033 (2018). 29652790 10.3390/plants7020033 PMC6027389

[25] Liang, Y., Chen, Q. I. N., Liu, Q., Zhang, W. & Ding, R. Exogenous silicon (Si) increases antioxidant enzyme activity and reduces lipid peroxidation in roots of salt-stressed barley (*Hordeum vulgare* L). *J. Plant. Physiol.* **160**, 1157–1164. 10.1078/0176-1617-01065 (2003). 14610884 10.1078/0176-1617-01065

[26] Daneshmand, B., Gholami, M., Etemadi, N. & Ehtemam, M. H. The water relation parameters are associated with the genotypic differences in the vase life of cut rose flowers. *Postharvest Biol. Technol.* **211**, 112829. 10.1016/j.postharvbio.2024.112829 (2024).

[27] Lichtenthaler, H. K. Chlorophyll fluorescence signatures of leaves during the autumnal chlorophyll breakdown. *J. Plant. Physiol.* **131**, 101–110. 10.1016/S0176-1617(87)80271-7 (1987).

[28] Hossein Hashemi, R., Nikbakht, A. & Aalipour, H. Enrichment of nutrient solution with oxygen nanobubble and foliar spraying of silicon nano chelate on some physiological characteristics in cut roses (Samurai and Jumilia). *J. Plant Proc. Func*. 13, 215–232 (2024). 10.22034/13.63.215

[29] Liu, B., Davies, K. & Hall, A. Silicon builds resilience in strawberry plants against both strawberry powdery mildew *Podosphaera aphanis* and two-spotted spider mites *Tetranychus urticae*. *PLoS One*. **15**, 0241151. 10.1371/journal.pone.0241151 (2020). 10.1371/journal.pone.0241151 PMC7723277 33290389

[30] Islam, T., Moore, B. D. & Johnson, S. N. Silicon suppresses a ubiquitous mite herbivore and promotes natural enemy attraction by altering plant volatile blends. *J. Pest Sci.* **95**, 423–434. 10.1007/s10340-021-01384-1 (2022).

[31] Ranger, C. M. et al. Influence of silicon on resistance of *Zinnia elegans* to *Myzus persicae* (Hemiptera: Aphididae). *Environ. Entomol.* **38**, 129–136. 10.1603/022.038.0116 (2009). 19791606 10.1603/022.038.0116

[32] Guerriero, G., Hausman, J. F. & Legay, S. Silicon and the plant extracellular matrix. *Front. Plant. Sci.* **7**, 463. 10.3389/fpls.2016.00463 (2016). 27148294 10.3389/fpls.2016.00463 PMC4828433

[33] Liang, Y., Sun, W., Zhu, Y. G. & Christie, P. Mechanisms of silicon-mediated alleviation of abiotic stresses in higher plants: a review. *Environ. Pollut*. **147**, 422–428. 10.1016/j.envpol.2006.06.008 (2007). 16996179 10.1016/j.envpol.2006.06.008

[34] Piotrowska-Niczyporuk, A., Bajguz, A., Zambrzycka, E. & Godlewska-Żyłkiewicz, B. Phytohormones as regulators of heavy metal biosorption and toxicity in green alga *Chlorella vulgaris* (Chlorophyceae). *Plant. Physiol. Biochem.* **52**, 52–65. 10.1016/j.plaphy.2011.11.009 (2012). 22305067 10.1016/j.plaphy.2011.11.009

[35] Savvas, D. & Ntatsi, G. Biostimulant activity of silicon in horticulture. *Sci. Hortic.* **196**, 66–81. 10.1016/j.scienta.2015.09.010 (2015).

[36] Li, Y. et al. Silica nanoparticles promote wheat growth by mediating hormones and sugar metabolism. *J. Nanobiotechnol*. **21**, 2. 10.1186/s12951-022-01753-7 (2023). 10.1186/s12951-022-01753-7 PMC9808955 36593514

[37] Wang, J. & Naser, N. Improved performance of carbon paste amperometric biosensors through the incorporation of fumed silica. *Electroanalysis* **6**, 571–575. 10.1002/elan.1140060707 (1994).

[38] Kaya, C., Tuna, L. & Higgs, D. Effect of silicon on plant growth and mineral nutrition of maize grown under water-stress conditions. *J. Plant. Nutr.* **29**, 1469–1480. 10.1080/01904160600837238 (2006).

[39] Greger, M., Landberg, T. & Vaculík, M. Silicon influences soil availability and accumulation of mineral nutrients in various plant species. *Plants* **7**, 41. 10.3390/plants7020041 (2018). 29783754 10.3390/plants7020041 PMC6027514

[40] Nozawa, S., Sato, T. & Otake, T. Effect of dissolved silica on immobilization of boron by magnesium oxide. *Minerals* **8**, 76. 10.3390/min8020076 (2018).

[41] Elsharkawy, M. M. & Mousa, K. M. Induction of systemic resistance against papaya ring spot virus (PRSV) and its vector *Myzus persicae* by *Penicillium simplicissimum* GP17-2 and silica (SiO2) nanopowder. *Int. J. Pest Manag*. **61**, 353–358. 10.1080/09670874.2015.1070930 (2015).

